# Improving quality of maternal and newborn care: An evaluation of enablers and barriers in implementing emergency obstetric and newborn care training in Bhutan and Lao People’s Democratic Republic

**DOI:** 10.1371/journal.pgph.0004584

**Published:** 2025-06-23

**Authors:** Sabera Turkmani, Kara Blackburn, Catherine Breen-Kamkong, Karma Tshering, Karma Choden, Alanya Chalernphon, Keodompone Vilivong, Rachel Smith, Caroline Homer

**Affiliations:** 1 Global Women’s and Newborns’ Health Working Group, Burnet Institute, Melbourne, Victoria, Australia; 2 Faculty of Health, University of Technology Sydney, Sydney, New South Wales, Australia; 3 Department of Sexual and Reproductive Health (SRH), UNFPA Asia-Pacific Regional Office (APRO), Bangkok, Thailand; 4 Sexual and Reproductive Health (SRH) Programme, UNFPA Bhutan Country Office, Thimphu, Bhutan; 5 Sexual and Reproductive Health (SRH) Programme, UNFPA Lao PDR Country Office, Vientiane, Lao People’s Democratic Republic; Jhpiego, UNITED STATES OF AMERICA

## Abstract

This study evaluates the effectiveness and sustainability of UNFPA-supported Emergency Obstetric and Newborn Care (EmONC) in-service training programs in Bhutan and Lao PDR, aiming to improve maternal and neonatal health services by enhancing health providers’ competencies in performing Basic EmONC signal functions. This study employed qualitative interviews with key informants and healthcare providers to identify enablers and barriers to the training program’s rollout and explore its sustainability. Overall, the EmONC training in Bhutan and Lao PDR was recognised as positive in enhancing the knowledge, skills, and confidence of healthcare providers and contributing to improved quality of care for women and newborns. Despite the successes, there are noted challenges such as financial and resource limitations, lack of alignment of policy and practice, legal barriers, insufficient mentoring and supportive supervision, and high workloads, which impeded the effectiveness and sustainability of the training. Future initiatives should focus on the long-term impact of EmONC training programs, examining how sustained policy reforms and regulatory alignment can empower healthcare providers to perform life-saving skills, ultimately improving maternal and newborn health outcomes.

## Introduction

Current progress to end preventable maternal and newborn mortality has stagnated and most countries will not meet Sustainable Development Goal (SDG) targets by 2030 [1]. In 2015, broad strategies to end preventable maternal and newborn deaths were agreed to in a global multi-partner initiative Ending Preventable Maternal Mortality (EPMM) and in the Every Newborn Action Plan (ENAP) [1,2]. Both global initiatives identified a common target of every birth being attended by skilled health personnel competent in the management of maternal and newborn emergencies [1]. Ensuring continued competence in the management of maternal and newborn emergencies requires significant investment in pre-and in-service education and training [1,2].

Emergency Obstetric and Newborn Care (EmONC) training is an effective evidence-based intervention to increase health care providers’ knowledge and skill in the management of maternity emergencies, and improve maternal and perinatal outcomes [3,4]. The majority of EmONC training programs are designed to prepare practitioners to perform the seven signal functions required for Basic Emergency Maternal and Newborn Care (BEmONC) (Table 1). However, effective implementation of EmONC training and service provision in low-resource settings is dependent upon investment in training and health service infrastructure, including availability of adequate resources [5]. Comprehensive assessments of EmONC training in low-resource settings indicate even with adequate infrastructure and resources, there remains a significant gap in staff competency to effectively perform Basic EmONC signal functions [6,7].

**Table 1:** Signal functions of basic (BEmONC) and comprehensive (CEmONC) emergency obstetric and newborn care [11].

BEmONC	CEmONC
1. Administration of parenteral antibiotic 2. Administration of parenteral anticonvulsants 3. Administration of parenteral uterotonics 4. Removal of retained products (manual vacuum aspiration) 5. Assisted vaginal delivery 6. Manual removal of the placenta 7. Resuscitation of the newborn	All of BEmONC functions plus: 8. Surgical capacity (caesarean section) 9. Blood transfusion

Causes of high maternal mortality rates in LMICs are multifactorial, evidence shows that lack of skills and knowledge of health care providers in Emergency Obstetric and Newborn Care (EmONC) is a key contributor to maternal mortality [8,9]. Competence in the management and skill in the signal functions of emergency obstetric and newborn care (EmONC) (Table 1) plays a critical role in preventing and managing life-threatening complications during pregnancy and childbirth [10,11].

Despite the global challenges in meeting the SDG targets, some countries have made excellent progress over the past decade [12]. In Bhutan, maternal mortality has decreased from 560 to 89 per 100,000 live births in 1990 and 2022 respectively [13] and in Lao PDR, from 650 deaths to 127 per 100 000 live births in 1995 and 2017 respectively [14]. Although significant reductions, neither country currently meets the 2030 SDG MMR target of 70/100,000 [13,14]. Both countries report considerable progress in coverage of skilled birth personnel attendance and a surge in women attending health institutions to give birth (Table 2). Key to these achievements in both countries have been the Ministries of Health prioritising capacity building of all maternity providers in essential obstetric signal functions and lifesaving skills (Table 1) [13,15]. In addition to commitment from both Governments, the United Nations Population Fund’s (UNFPA) Maternal and Newborn Health Thematic Fund (MHTF) targeted support of the International Conference on Population and Development (ICPD) Call to Action in ending preventable maternal death has contributed to accelerating progress towards reducing maternal mortality in both Lao PDR and Bhutan [16].

**Table 2:** Characteristics of countries [13,15].

Country	Bhutan	Lao
**Health system structure & EmONC services**	• Bhutan operates a three-tiered, publicly financed and managed healthcare system. This includes 186 primary health centres (PHCs) at the primary level, 54 hospitals at the secondary level (district hospitals), and 3 referral hospitals at the tertiary level. Healthcare services are provided free of charge at all levels to citizens. • Basic EmONC services are available at all hospitals and primary health centres. Comprehensive EmONC services are provided at 3 referral hospitals and 5 district hospitals. • Country-wide assessment of EmONC services reported that there is a shortage of key staff (40% medical doctors and 45% of nurse midwives)	There are four levels of maternity services in the health system structure: • Central level (5 central hospitals, 3 central centres • Provincial level (17 provincial hospitals) • District level (135 District Hospital (34 Type A and 101 Type B) • Health centres (1072) (MoH data 2023) EmONC (including basic and comprehensive) implemented across all levels of health system
**Midwifery education system**	• Bhutan has four institutions (1 public, 3 private) that produce approximately 150 nurse-midwives annually. • BEmONC training is sponsored by UNFPA in collaboration with the Ministry of Health. The program includes seven essential life-saving skills and provides Champion Training to mentor other healthcare providers. • Integrated into both pre-service and in-service education programs. It offers a 3-year diploma and a 4-year bachelor’s degree. Graduates are registered with the Medical and Health Professional Council, qualifying them to serve as both nurses and midwives in clinical settings. • Nurse-midwives predominantly work in hospitals, while health assistants serve in primary care settings. Emergency Medical Responders (EMRs) are also deployed in hospitals. • All graduates (nurses, midwives, EMRs, and health assistants) are trained to provide essential skills, including delivery assistance, newborn resuscitation, postpartum hemorrhage (PPH) management, and tear repair.	• Two midwife education programs exist, these are a 24-month Bachelor’s program and a 36-month Higher Diploma program. • Midwives, based on their scope of practice, are allowed to provide BEmONC services. • Midwives who are trained in the BEmONC are expected to perform the seven essential life-saving signal functions and also to acquire the skills to become master trainers and mentors to train other nurse midwives in BEmONC through a supportive approach known as Champion Training. The MOH, ensures the adequate capacity of the midwifery workforce by conducting emergency obstetric training for midwives to strengthen their clinical knowledge and skills, enabling them to manage the major causes of maternal deaths in Lao

UNFPA supported the implementation and roll-out of the Jhpiego Helping Mothers Survive (HMS) and the American Academy of Pediatrics Helping Babies Breathe (HBB) training programs in Lao PDR and Bhutan. The HMS training methodology involved an initial series of Master training, and a supported stepdown approach to create a pool of Master trainers and thus allow for broader roll-out the training. The HMS/HBB methodology encourages all participants to engage in regular short practise sessions (low-dose, high-frequency) in their workplace, for the intended purpose of promoting retention and enhancement of participants’ knowledge and skills [17]. Following initial Master training in 2018–2019 where 12 (Bhutan) and 7 (Lao PDR) Master trainers were produced, an additional 212 health providers in Bhutan, and 187 health providers in Lao PDR had completed the HMS/HBB EmONC training program at the time of this evaluation.

This study was developed to evaluate the UNFPA-supported EmONC training programs in Lao PDR and Bhutan.. These countries were identified by UNFPA as appropriate settings for the evaluation due to their distinct health systems. Despite of diverse contextual factors, the findings provide valuable insights into how EmONC services can be scaled and implemented across varied healthcare environments in LMICs.

The aim of the study was to identify enablers and barriers to the roll-out of the EmONC training program in these settings and explore its sustainability, contributing to better understanding of how such interventions can be improved or adapted to different resource-limited countries.

## Method

### Ethics statement

Ethical approval was obtained from the Research Ethics Board for Health, Ministry of Health, Thimphu, Bhutan (Ref No. ERRHM/PER-03/2023–24/1460) and Lao PDR Ministry of Health, National Ethics Committee for Health Research (NECHR) (Ref No. 57/NECHR). Potential participants were provided with information on the project and were invited to ask questions for clarification, and written consent was obtained.

### Design

The study used a multi-method design using qualitative and quantitative approaches, and this paper presents the qualitative findings. The qualitative evaluation aimed to identify enablers and barriers to the roll-out of the training program in these settings and is the focus of this paper.

### Data collection

Qualitative data were collected through a series of one-to-one, semi-structured interviews with key informants (KI) who were engaged in EmONC related decision making (Ministry of Health personnel, District Health Authorities personnel and trainers) and, with the healthcare providers (HCPs) who participated in the training program, primarily midwives, as well as nurses and health assistants involved in the provision of maternity services.

Participants were recruited purposively through the Ministry of Health and UNFPA country offices in Bhutan from 01/11/2023- 18/12/2023 and in Lao PDR from 18/11/2023 to 13/01/2024. A local research consultant was independently recruited through a transparent and open job advertisement in each country and conducted interviews with each participant in a convenient location, which for the majority was the private settings within health facilities or workplaces and interviews conducted in participants’ preferred local languages, using a semi-structured guide (Annexe 1). The interviews were audio recorded, transcribed verbatim, transcribed into English, de-identified and stored securely in a password-protected cloud service.

### Data analysis

We employed framework analysis, a systematic method well utilised in healthcare research [18]. This technique combines predetermined codes from the literature, which were based on key themes relevant to the study’s focus, such as ‘ barriers and facilitators of EmONC’, ‘areas for improvement’ ‘EmONC services and ‘sustainability of EmONC’, along with additional codes emerged from the data, such as the ‘significance of mentorship’ and ‘country-specific challenges’, ensuring that the findings are aligned with the research goals and lead to actionable recommendations [19]. NVivo software facilitated the final data organisation, guiding us through the crucial steps of familiarisation, summarisation/interpretation, and the identification of codes and themes.

## Findings

Twenty-one qualitative interviews were undertaken in Bhutan, and nine in Lao PDR. Participants were selected from a mix of rural and urban areas in both countries and included representatives from the Ministry of Health, education institutes, facilities that provide EmONC services and donor agencies. (Table 3).

**Table 3:** Number of interviews by countries.

Interviews	Key Informants (KI)	Healthcare Providers (HCP)	Total
Bhutan	10	11	21
Lao PDR	4	5	9

Findings are presented in line with the aim of the research to identify enablers, barriers and/or challenges to the roll-out and sustainability of the EmONC training program in Bhutan and Lao PDR. The results are categorised under the key areas of implementation, and the challenges and barriers encountered during the initiation and subsequent roll-out of the EmONC training program.

### Implementation of the EmONC training

HCPs in both countries highlighted the benefits of a well-structured design approach to the EmONC training, emphasising the division of content to ensure participants received a balanced education program that addressed both theoretical and practical aspects of emergency management. Participants described beneficial aspects of the training as follows:

*Each module had a theoretical and a practical component, but we emphasised the theoretical part by using a round-table approach to enhance understanding and retention (HCP, Lao PDR).*

*The fact that LDHF EmONC is not just theoretical knowledge that we gain from these modules, we do have more of hands-on skill practice and at the last we have to pass through various OSCEs in order to be competent in managing complications (HCP, Bhutan).*

Key Informants felt regular monitoring and assessments of knowledge as part of the EmONC implementation, alongside some skill monitoring through on the job mock assessments tailored to specific emergency scenarios was beneficial for learning and subsequent care delivery. However, they also raised concerns about the absence of formal monitoring of the practical aspects of the EmONC training and suggested that systematic assessment of these aspects could significantly aid in retaining knowledge and skills to improve the impact of the training program. Participants explained the following:

*We do some form of assessment, but somehow, we can say that yes in terms of knowledge gaining yes, they have gained, even practical because during the session they had to perform practical because we have conducted OSCE also, … but in reality, in real situation, how things are happening - we really don’t know (KI, Bhutan).*

*…we also periodically monitor the progress and quality of the care and provide assistance in case if [sic] any issues in competencies. …Periodic visits from trainers and provincial health staff to assess and support the trainees, taking advantage of the field work and monitoring of midwife could be a way to assess them (KI, Lao PDR).*

Implementation support with ongoing staff training and transfer of knowledge and skills was considered crucial, alongside a focus on ensuring adequate supervision within healthcare facilities. The importance of orientation and training for new staff in the maternity ward was emphasised as a national priority to enhance maternal and neonatal outcomes. Participants described how national policies and goals were used to support the implementation process:

*…it is our role and responsibility as a EmONC focal [point] to deliver our knowledge and updated skills to all the health care staff to just achieve our APA [Annual Performance Agreement, national goals to bring our maternal and neonatal death [down] by 2025 (KI, Bhutan).*

*EmONC training has been integrated into the existing healthcare system and policies in several ways, …aligning it with the national strategy of integrating mother, child health and reproductive health, and incorporating EmONC modules into pre-service and in-service education curricula for various cadres of health workers (KI, Lao).*

Access to and utilisation of evidence-based clinical standards and competency guidelines were identified as a critical enabling factor for timely management of emergency situations and improvement in outcomes. Participants identified a need for strong emphasis on clear protocols and guidelines describing the roles and responsibilities of midwives and nurses when performing specific emergency procedures. Key informants in both countries described the need as follows:

*To ensure the sustainability of the knowledge and skills acquired through EmONC training, several measures have been taken at different levels. Firstly, the midwives have access to the updated version of the scope, clinical standards and national competency based on ICM 2019, which defines the expected level of performance and quality of care for midwifery services (KI, Lao PDR).*

*When we do have written protocol for that [performing emergency skills], we don’t have to wait for doctor…. For instance, in case of PPH we provide care [independently] and do not wait for the doctors to come…as we have PPH management protocol. [KI, Bhutan]*

### Challenges and barriers of the EmONC training

Overall, there were challenges and barriers in implementing EmONC training effectively in Lao PDR and Bhutan. The challenges included systemic barriers, including financial and other resource limitations, regulatory challenges, and logistical and geographical barriers. Inadequate training infrastructure and scarcity of necessary resources, such as limited suitable training environments, insufficient training materials, and lack of access to continued professional development, were recurring issues. The narratives also outlined the dynamic tension between the impactful results of the training and the underlying vulnerabilities that could compromise its sustainability and expansion, suggesting a need for a resilient framework that continuously adapts to and addresses the complex challenges.

One participant identified issues in monitoring the training impact and geographical challenges associated with monitoring:

*… the training is not continuous and there is no system for monitoring the performance and outcomes of EmONC. Some areas in my province are inaccessible during the rainy season, which further limits the monitoring of EmONC (KI, Lao).*

Participants highlighted critical gaps in ensuring all healthcare providers are uniformly equipped with current EmONC knowledge and skills. The predominant challenge was the lack of ongoing and refresher training for maintenance of competence. Participants noted a gap in continuous learning, with some respondents not having received EmONC training and others acknowledging the need for periodic updates to maintain their skills. One participant explained the need for updates:

*Somehow, we have to maintain but we need to explore, but in this regard if there are new updates … and all then I think we need to have certain refresher course or something like that… because after giving training if nothing is done then maybe we are not doing anything correctly (HCP, Bhutan).*

Participants also identified a need to balance access to training with clinical work demands. These access issues were compounded by logistical challenges in organising and delivering the training program, particularly in provincial or more remote areas. The need for consideration of service demands is evident in this participants feedback:

*…it is a whole day training know, so it will disturb our work station … the work duties, so have to arrange separate like two training like on turn wise basis, so that we don’t get disturbed in between. It is very difficult to arrange those training, so that’s the drawback (HCP, Bhutan)*

With the following quote outlining some of the organisational challenges and barriers:

*One of the challenges we faced was the communication gap between the central organizers and the provincial trainees. Sometimes, the trainees did not receive the notice for the training and did not show up.(KI, Lao)*

System challenges identified included a lack of access to resources, inadequate infrastructure, high staff turnover, and logistical and financial constraints. In addition to the challenges and barriers to implementation of the training program, healthcare providers commented on system-based challenges such as a lack of availability of essential medications in healthcare facilities which impacted the ability to effectively manage emergencies and perform EmONC signal functions as evidenced below:

*I have encountered some challenges in obtaining the necessary resources, equipment, or support to implement the EmONC practices learned during the training. For example, sometimes the medication is out of stock, or the referral system is not functioning well. (HCP, Lao)*

Furthermore, participants reported constraints that hindered the effective application of the EmONC training skills. This included lack of accepted protocols, staff shortages and heavy workloads, which limited the opportunity for healthcare providers to fully apply their knowledge and skills. One health care provider explained it like this:

*… sometimes we have the protocol and all things written there but then we don’t have the permission or equipment, so then it’s very difficult, sometimes we don’t have other staff, you know the ward is so busy and then we cannot have our colleagues here… so, because of HR issues also (HCP, Bhutan).*

Challenges related to the lack of practical training resources, like simulation models in hospitals, impacted the hands-on training experience. Healthcare providers described difficulties in implementing EmONC LDHF practise sessions due to the unavailability of equipment, impacting the quality of the ongoing training sessions. For example:

*Usually when it comes to the resources, we don’t have those … like exact mannequins… Mama Natalie… those things, so in the first training I just improvised with made cartoon box [sic], usually like other training we have to like for first aid even for advanced neonatal resuscitations… those things we have to follow the teaching aids from the faculties, so it’s also difficult to get here (KI, Bhutan).*

*The lack of equipment also limits our ability to demonstrate some of the skills or techniques that we learned in the training (HCP, Lao).*

In Lao PDR, the lack of clear and comprehensive guidelines or protocols to guide the emergency response, particularly for drug administration, were identified as barriers to providing quality emergency management and care:

*The challenges that I faced in implementing the practices or techniques learned in the EmONC training was the clarity of the guideline. The guideline was translated from English to Lao, but some parts were not clear, and the trainer did not understand them well (HCP, Lao).*

Some participants reported concern regarding the rarity of specific obstetric emergencies and the lack of legal authorisation that may prevent healthcare providers from applying effective management, and they identified that this may led to a lack of confidence in managing infrequent emergencies:

*I am also very doubtful with the skills because we get [the cases] very rarely … in a year we get 30-35 delivery cases and while sometimes we get at the max 3-4 deliveries in a month we get hands on, but then again following month if we don’t get cases then we tend to lose our skills… Especially Pre-Eclampsia and Eclampsia its very rare… in 2020 or 2021 we saw just one... But still there is vast difference between before we started taking classes [EmONC training]and after (HCP, Bhutan).*

Such lack of confidence was also evident among HCPs in Lao as described by this participant:

*The EmONC training covered various topics and areas that I found relevant and applicable to my clinical practice. However, I still have some concerns about providing some services in case of emergency, especially delivering the [retained] placenta and using the balloon tamponade (HCP, Lao).*

Some HCPs highlighted the gap between the national regulatory framework, scope of practice, and the practical realities faced by midwives in both Bhutan and Lao PDR in terms of their authorisation for performing EmONC functions:

*…the regulation says we can’t do it but the circumstances demand it sometimes… so I think at that time we have to intervene…but higher authority people are there and they said that nurses [Midwives] cannot do vacuum extraction because of regulation facts… but if we are trained and have certificate then that will support us (HCP, Bhutan).*

*…we need to receive more support and guidance from the higher-level authorities on the scope and responsibilities of midwives. Sometimes, there is confusion or conflict about what we can or cannot do as midwives, which can affect our confidence and performance. (HCP, Lao)*

The narrative highlights an evaluation of the positive outcomes of EmONC training and existing challenges that may affect its ongoing effectiveness and sustainability of the program:

*My experience has been rewarding and challenging at the same time…, I have faced some difficulties and barriers in implementing the [EmONC] training, such as lack of resources, inadequate infrastructure, high turnover of staff, and low motivation of some trainees. These challenges require constant monitoring, evaluation, and follow-up to ensure the sustainability and effectiveness of the training program.(KI, Lao)*

## Discussion

Overall, the EmONC training in Bhutan and Lao PDR was recognised as positive in enhancing the knowledge, skills, and confidence of healthcare providers and, in turn, potentially contributing to improved quality of care and health outcomes for women and babies. Participants appreciated the hands-on approach and practical exercises, which helped them bridge the gap between theory and practical training exercises that directly applied to real-world scenarios. Despite the successes, there are noted challenges such as financial and resource limitations, practical and legal barriers, insufficient mentoring and supportive supervision, and high workloads, which impeded the effectiveness and sustainability of the training.

Ensuring the long-term sustainability of EmONC programs, such as the Helping Mothers Survive (HMS) and Helping Babies Breathe (HBB), poses significant challenges [20]. Previous studies in LMICs, align with the findings in our research, indicating that without a supportive health system, adequately trained workforce, financial resources, essential training materials and incentives for participants, the potential benefits of training programs are significantly limited [20–22]. The absence of administrative backing for the training and subsequent national rollouts hinders the smooth integration of training programs into existing healthcare services and negatively impacts the program’s effectiveness.

The scarcity of infrastructure and practical resources, such as simulation models and equipment, has been identified as a barrier to the success of the EmONC training experience. Both training and clinical resource shortages emerged as a common challenge, with reported deficiencies in essential drugs and access to training equipment [23]. Additionally, in this study, similar to other research, healthcare providers struggled to fully engage in the practice of EmONC functions as part of training due to demanding clinical duties and workloads and the inability to allocate sufficient time for learning. This issue highlights a broader systemic problem within healthcare systems, where providers are often juggling excessive patient volumes and administrative tasks alongside their professional development endeavours [24,25].

Research indicates that regular, hands-on training is more effective than traditional, infrequent educational interventions [23]. The lack of ongoing supervision and refresher training reported in our study leaves healthcare providers vulnerable to skill erosion and outdated knowledge, which may lead to poorer health outcomes [3,24]. Evidence suggests that continuous learning along with supportive mentoring and regular refreshers training are essential for retaining skills and competencies in EmONC, ensuring healthcare providers remain prepared to manage emergencies and deliver the highest standard of care [24].

To maintain competence in managing obstetric and newborn emergencies, an evidence-based approach utilising standardised guidelines and protocols is crucial in enhancing decision-making and improving the quality of maternal and newborn outcomes [26]. Our study in Bhutan and Lao PDR highlights the importance of access to clear clinical guidelines and regulatory support for the successful implementation and sustainability of EmONC programs. Aligning with standardised processes has been identified as a key enabler in quality improvement within resource-limited environments, contributing significantly to the overall effectiveness of maternal and newborn care [27].

To reduce maternal mortality, the Sustainable Development Goals (SDGs) 2030 emphasize the importance of a supportive environment where midwives are legally authorized and supported to deliver EmONC services [1]. Correspondent to findings from similar contexts, our study found a lack of alignment and discrepancies between international standards and local policy and legal authorisations for practice hinders midwives’ ability to perform essential EmONC signal functions and potentially impacts the timely management of emergency cases [28]. Evidence suggests legal and regulatory review to ensure that midwives as frontline healthcare providers are legally authorised and empowered to perform the essential competencies of basic emergency obstetric and newborn care provision [29]. Moreover, evidence indicates that a strong alignment between legislation and clinical practice significantly enhances the quality of care [29,30]. By implementing legislative reforms, investing in education, defining a clear scope of practice for midwives in line with international standards, allocating adequate resources, and engaging communities, governments can move towards improving maternal and newborn health [1,31]. However, despite such reforms being incorporated into the Lao PDR national plan for midwifery improvement in 2016, our findings suggest a shortfall in the enforcement of such strategies [32].

### Strengths and limitations

This study is one of the few evaluations examining the effects of EmONC training in Bhutan and Lao PDR. The results contribute valuable data to the limited body of knowledge in this area, enhancing the understanding of EmONC training in low-resource contexts. Moreover, the study provides crucial insights that could inform policy and practice, particularly in developing the structure and delivery of EmONC training programs in LMICs and establishes a baseline for future research in the field.

The study did not explore variations in EmONC in-service training provided by different organisations, nor did it examine the differences in health facility structures, national regulations, and practices. A further study based on health facilities’ structure, rural versus urban settings, and work experience might yield different results.

## Conclusion

The success and challenges of the EmONC training program in Bhutan and Lao PDR illustrate a transformative approach to enhancing maternal and newborn healthcare quality, especially in low and middle-income countries. The resource constraints and policy barriers underscore the complexity of implementing and sustaining such initiatives in resource-limited settings, highlighting the need for a holistic approach that includes training, adequate infrastructure, supportive supervision, and conducive policy environments.

Future initiatives should focus on the long-term impact of EmONC training programs, examining how sustained policy reforms and regulatory alignment can empower healthcare providers to perform life-saving skills, ultimately improving maternal and newborn health outcomes. Effective collaboration among diverse stakeholders, including local governments, national and international partners, and professional associations, is essential for enhancing the quality of maternal and child healthcare services. Additionally, it is crucial to explore how such initiatives can be effectively scaled and replicated in other similar contexts while considering each setting’s unique challenges and resources.

## Supporting information

S1 Checklist Inclusivity in global research. (DOCX)
